# The Abscission Checkpoint: A Guardian of Chromosomal Stability

**DOI:** 10.3390/cells10123350

**Published:** 2021-11-29

**Authors:** Eleni Petsalaki, George Zachos

**Affiliations:** Department of Biology, University of Crete, Vassilika Vouton, 70013 Heraklion, Greece; grad600@edu.biology.uoc.gr

**Keywords:** chromatin bridges, abscission checkpoint, cytokinesis, actin patches, midbody, DNA damage, chromosomal instability, Aurora B, Chk2, ATM, CPC, Chmp4c, ESCRT, cancer

## Abstract

The abscission checkpoint contributes to the fidelity of chromosome segregation by delaying completion of cytokinesis (abscission) when there is chromatin lagging in the intercellular bridge between dividing cells. Although additional triggers of an abscission checkpoint-delay have been described, including nuclear pore defects, replication stress or high intercellular bridge tension, this review will focus only on chromatin bridges. In the presence of such abnormal chromosomal tethers in mammalian cells, the abscission checkpoint requires proper localization and optimal kinase activity of the Chromosomal Passenger Complex (CPC)-catalytic subunit Aurora B at the midbody and culminates in the inhibition of Endosomal Sorting Complex Required for Transport-III (ESCRT-III) components at the abscission site to delay the final cut. Furthermore, cells with an active checkpoint stabilize the narrow cytoplasmic canal that connects the two daughter cells until the chromatin bridges are resolved. Unsuccessful resolution of chromatin bridges in checkpoint-deficient cells or in cells with unstable intercellular canals can lead to chromatin bridge breakage or tetraploidization by regression of the cleavage furrow. In turn, these outcomes can lead to accumulation of DNA damage, chromothripsis, generation of hypermutation clusters and chromosomal instability, which are associated with cancer formation or progression. Recently, many important questions regarding the mechanisms of the abscission checkpoint have been investigated, such as how the presence of chromatin bridges is signaled to the CPC, how Aurora B localization and kinase activity is regulated in late midbodies, the signaling pathways by which Aurora B implements the abscission delay, and how the actin cytoskeleton is remodeled to stabilize intercellular canals with DNA bridges. Here, we review recent progress toward understanding the mechanisms of the abscission checkpoint and its role in guarding genome integrity at the chromosome level, and consider its potential implications for cancer therapy.

## 1. Introduction

To ensure accurate distribution of the genetic material from the parent to the two daughter cells during cell division, completion of cytokinesis (abscission) is tightly coordinated with chromosome segregation [1]. In response to chromosome segregation defects giving rise to lagging chromosomes or chromatin bridges, i.e., strings of missegregated chromatin connecting the anaphase poles or daughter nuclei [2], eukaryotic cells delay abscission to prevent chromatin breakage or tetraploidization by regression of the cleavage furrow [3,4,5,6,7].

An abscission delay in response to anaphase defects was first identified in budding yeast and called “NoCut” [3]. It was later shown that chromatin trapped inside the midzone is the primary source of NoCut [8]. NoCut depends on the catalytic activity of Ipl1/Aurora kinase and requires targeting of the chromosomal passenger complex to the central spindle in anaphase [3,8]. In mammalian cells, the abscission delay in response to chromosome segregation defects is called the “abscission checkpoint” and is dependent on Aurora B kinase activity at the midbody [4,5,7,9]. Impaired abscission checkpoint signaling can lead to accumulation of DNA damage, micronuclei formation or polyploidy which are associated with tumourigenesis and tumour evolution [10,11,12,13].

In the last decade, additional triggers of the abscission checkpoint, such as nuclear pore defects [14], DNA replication stress [15] or high intercellular bridge tension [16] have been identified, and signaling pathways of the abscission checkpoint have been uncovered (for previous reviews see [17,18]). In this review, we focus on abscission checkpoint mechanisms in response to chromatin bridges. More specifically, we review current literature on the mechanisms that regulate Aurora B localization and catalytic activity at the midbody, and describe signaling pathways downstream of Aurora B that impose the abscission delay in mammalian cells. We also describe mechanisms that stabilize chromatin bridges and intercellular canals during the abscission delay, discuss the significance of the abscission checkpoint for maintaining genome stability and consider how abscission checkpoint proteins can be targeted to potentially improve cancer therapy.

## 2. A Mechanistic Model of Abscission

**Figure 1:**
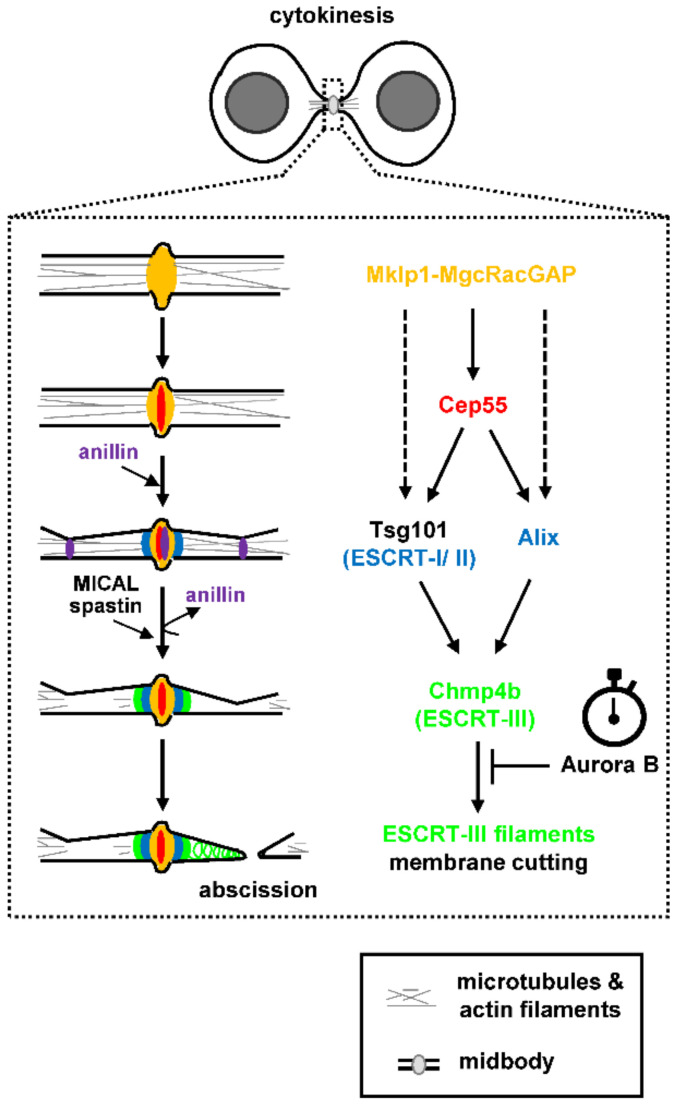
Molecular mechanisms of abscission. Molecular pathways that lead to membrane cutting and clearance of microtubules/actin filaments from the abscission site. See text for details. Potential mechanisms of Tsg101 and Alix recruitment to the midbody that are independent of Cep55 are indicated by dashed arrows. The clock symbol indicates the role of Aurora B as an abscission timer.

Abscission, the final step of cytokinesis during which the narrow intercellular canal that connects the two daughter cells is cleaved, requires plasma membrane remodeling at the constriction sites as well as reorganization of the cytoskeleton inside the intercellular canal (reviewed in [1,19]). The evolutionarily conserved Endosomal Sorting Complex Required for Transport (ESCRT) machinery that constricts and cuts membranes during multivesicular body formation, viral budding or nuclear envelope reassembly after mitotic exit, also delivers the membrane scission step during abscission (reviewed in [20,21,22]). The ESCRT machinery comprises the ESCRT-I, II and III modules and, at the later stages of cytokinesis, ESCRT proteins are positioned at the midbody, a microtubule-dense structure inside the intercellular canal that serves as platform for the assembly of the abscission machinery [23]. In mammalian cells, the microtubule bundling protein Cep55 associates with the Mklp1-MgcRacGAP (centralspindlin) complex at the midbody; in turn, Cep55 recruits the ESCRT-I component Tsg101 and the ESCRT-associated protein Alix to the midbody (Figure 1; [24,25,26,27]). Tsg101 interacts with other ESCRT-I/II components to recruit ESCRT-III members at the midbody, of which Chmp2a, Chmp4b and IST1 proteins are main constituents, to deliver the final cut [28,29,30]. Furthermore, Tsg101 interacts with septin-9 and septin-9 promotes formation of ESCRT-III rings at the midbody and their expansion into cones (helices) at the abscission site that correlates with abscission [31,32]. The ESCRT-associated protein Alix on the other hand, acts in parallel to the Tsg101-ESCRT-I/II pathway to recruit ESCRT-III proteins to the midbody [30]. In addition, recent studies have shown that in *Cep55*-knockout mice, Tsg101, Alix, and ESCRT-III can still be recruited at the midbody (although at reduced levels) compared with controls and that abscission (although delayed) can be completed in many cell types, suggesting abscission can also proceed via Cep55-independent mechanisms (Figure 1; [33,34]). Interestingly, in Drosophila which has no Cep55, Alix and Tsg101 recruitment to the midbody is promoted by the centralspindlin component Pavarotti (the human Mklp1 homologue; [35]). Furthermore, localization of the ESCRT-I component AKTIP to the midbody depends on Mklp1 but is independent of Cep55 in human cells [36].

Cep55 and Tsg101 form circular structures in the central area of the midbody whereas Alix, ESCRT-II (e.g., Vps36) and ESCRT-III (e.g., Chmp6, Chmp2a, Chmp4b, IST1) subunits form double rings next to the central midbody as determined by high-resolution imaging [28,31,37,38,39]. In later stages of cytokinesis, ESCRT-III polymers containing Chmp4b and IST1 form spiral structures with progressively smaller diameters at the secondary ingression site (that will become the abscission site) at approximately 1 μm distance from the midbody (Figure 1; [37,38,39]). This reorganization of ESCRT-III into spirals is thought to promote membrane deformation and scission at the abscission site and requires ESCRT-binding to the ATPase Vps4, which promotes remodeling of the ESCRT-III filaments by subunit turnover [37,38,40,41,42]. The Aurora B kinase acts as “abscission timer” in normally segregating cells by inhibiting proper localization and function of Vps4 at the midbody as described below and a reduction in Aurora B catalytic activity is required for abscission (Figure 1; [4,5,6]). It was also recently shown that ESCRT-III localization at the abscission site depends on a tripartite module comprising Alix, the transmembrane proteoglycan syndecan-4, and syntenin [43]. It is proposed that Alix-syntenin anchors ESCRT-III to the membrane, while syndecan-4 stabilizes ESCRT-III polymers at the abscission site [43].

Maturation of the intercellular canal and formation of the secondary ingression site precede assembly of ESCRT-III filaments at the abscission site. The anillin-septin cytoskeleton makes an initial ingression and anillin rings are detected at the midbody and future secondary ingression sites (Figure 1; [32]). Those anillin rings dissipate before Chmp4b recruitment to the midbody; furthermore, anillin-dependent recruitment of septin-9 to the intercellular canal is required for Chmp4b localization to the abscission site [32,44]. The narrowing of the intercellular canal from ~2 μm to ~100 nm (secondary ingression) is also mediated by fusion of Rab11/FIP3-positive recycling endosomes; however, the molecular events that specify positioning of the secondary ingression site require further investigation [45,46].

To allow membrane constriction and scission by the ESCRT machinery, polymerized actin (F-actin) is removed from the abscission site by several known mechanisms (reviewed in [19]). First, Rab35 GTPase recruits the PtdIns(4,5)P2 lipid phosphatase OCRL to the intercellular canal; in turn, hydrolysis of plasma membrane phosphoinosides by OCRL restricts F-actin oligomerization and promotes abscission [47]. Second, Rab11/FIP3-positive endosomes deliver the p50RhoGAP cargo to the intercellular canal to limit Rho GTPase activation and actin polymerization [46]. Third, actin capping protein, a protein that binds to actin “barbed end” where monomer addition predominantly occurs, is transported to the intercellular canal to counteract formin-based generation of actin filaments [48]. Fourth, cells induce depolymerization of existing actin filaments through Rab35 GTPase and MICAL1 oxidoreductase, an enzyme that oxidizes methionine residues on F-actin and induces filament depolymerization (Figure 1; [49]). Rab35-binding is sufficient to activate the enzymatic activity of MICAL1 towards actin filaments in vitro; furthermore, Rab35 recruits MICAL1 close to the abscission site to clear polymerized actin before abscission [49].

In addition to F-actin, cells must clear microtubules at the secondary ingression site before canal cleavage. For this purpose, the microtubule-severing AAA ATPase spastin directly interacts with the ESCRT-III component Chmp1b and is recruited to the future abscission site to coordinate membrane cutting with microtubule severing (Figure 1; [50,51]). Localized microtubule buckling and breaking may also contribute to microtubule severing at the secondary ingression site [45].

## 3. Abscission Checkpoint Mechanisms in Cytokinesis with Chromatin Bridges

Origins of DNA Bridges

Anaphase DNA bridges, i.e., threads of DNA stretching between the two segregating chromosome masses, can result from unresolved homologous recombination intermediates, incomplete DNA replication, chromosome catenation, or an attempt to segregate dicentric chromosomes that result from telomere-to-telomere fusion (reviewed in [52]). DNA bridges increase in the presence of DNA replication, decatenation or condensation inhibitors, but are also observed even in the absence of exogenous stress, especially at centromeres in mammalian cells [53,54,55]. In contrast to the other classes of DNA bridges that can occur during normal DNA metabolism, telomere fusions are triggered by telomere dysfunction such as excessive telomere shortening or dysregulation of the shelterin complex [56,57]. Furthermore, anaphase bridges induced by DNA replication stress, decatenation or condensation defects, but not dicentric chromosomes, delay abscission in budding yeast, showing the molecular origin of DNA bridges is important for the activation of NoCut [58].

DNA bridges can be also classified into (“ordinary” or “bulky”) chromatin bridges that stain with standard DNA dyes such as DAPI, Hoechst, etc, and ultrafine bridges that are so subtle they are practically invisible by conventional DNA dyes and can be only visualized by staining for nuclear membrane proteins such as LAP2, or DNA helicases such as PICH and BLM that localize to those bridges [53,54,59]. Spontaneous ultrafine anaphase bridges typically arise from double-stranded DNA catenates at centromeres; this type of ultrafine bridges exists in every mitosis and is characterized by the association of centromeric markers (e.g., CENP-A or Hec1) at the bridges’ termini [53,54,60,61]. Ultrafine bridges can also arise from late replication intermediates at common fragile sites where replication is often delayed [55,62], persistent DNA catenanes at ribosomal DNA loci [63], or from unresolved DNA intermediates that are generated from homologous recombination [64,65]. Chromatin bridges, on the other hand, are relatively rare under unperturbed conditions and are mostly caused by unresolved recombination intermediates in yeast cells [66]. We will mainly refer to abscission checkpoint mechanisms in response to spontaneous or stress-induced (bulky) chromatin bridges in mammalian cells. However, it was recently shown that treatment of cells with a topoisomerase inhibitor that promotes formation of ultrafine bridges delays abscission, suggesting the abscission checkpoint can also be activated by ultrafine bridges persisting in telophase in human cells [67].

## 4. The Aurora B Kinase

In eukaryotic cells, the abscission checkpoint requires persistent localization and catalytic activity of the conserved kinase Aurora B at the midbody [3,4,8]. Aurora B is the catalytic subunit of the Chromosomal Passenger Complex (CPC) also comprising the scaffolding protein INCENP and the nonenzymatic subunits Survivin and Borealin (reviewed in [68,69,70]). The CPC is required for several mitotic processes including chromatin condensation, centromere cohesion, kinetochore-microtubule attachment, regulation of the mitotic spindle checkpoint, cleavage furrow ingression, and the regulation of abscission [68,69,70].

### 4.1. Aurora B Localization to the Midbody

Aurora B binds to the C-terminal region of INCENP called the “IN-box” (amino acids 892-900 of human INCENP) and this interaction is essential for proper Aurora B localization and kinase activity [71,72]. From late prophase until metaphase, the CPC localizes to the centromere through INCENP-interactions with the phosphorylated histones H3-threonine 3 and H2A-threonine 120, which are mediated by Survivin and Borealin (reviewed in [73]). Furthermore, a relatively small population of catalytically active Aurora B is also detectable at prometaphase kinetochores where it phosphorylates kinetochore substrates in human cells [74,75,76]. Soon after anaphase onset, the CPC translocates to the central spindle, before localizing to the midbody in telophase to regulate abscission. CPC-translocation to the central spindle requires dephosphorylation of histone H2A-threonine 3 and of Cdk1-target residues inside INCENP and Mklp2 [77,78,79,80]. Dephosphorylation of histone H2A-threonine 3 reduces INCENP-affinity for centromeres and enhances the electrostatic interactions of a putative coiled-coil domain of INCENP with midzone microtubules by promoting CPC-multimerization [79]. Furthermore, dephosphorylation of the Cdk1-target site INCENP-threonine 59 acts as a switch to promote INCENP-association with the Mklp2 kinesin that directly binds to microtubules and increase the microtubule-dependent ATPase activity of Mklp2, thus driving CPC localization to central spindle microtubules [80].

**Figure 2:**
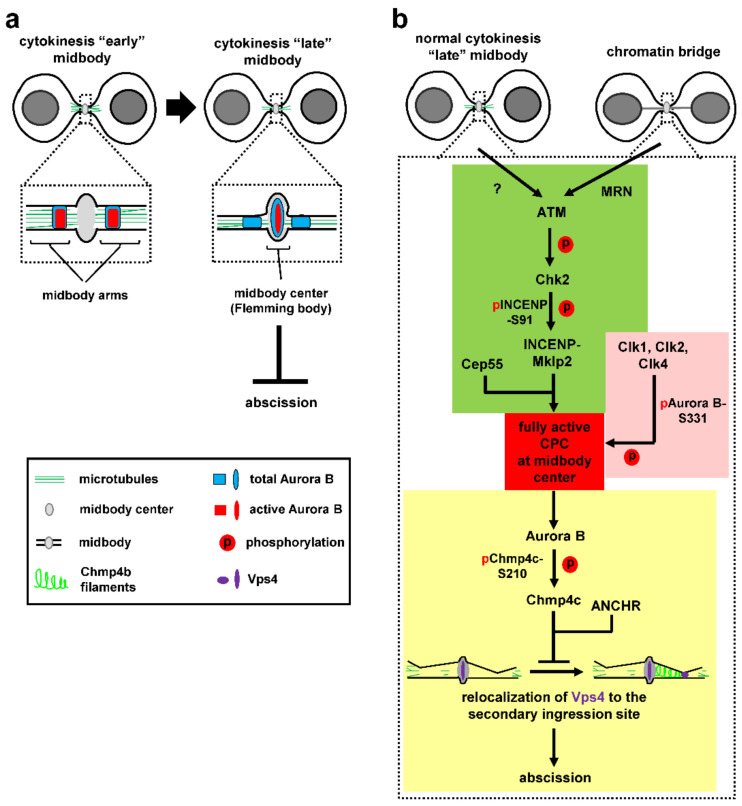
Abscission checkpoint pathways in higher eukaryotic cells. (**a**) Active Aurora B localizes inside the midbody center to delay abscission in late midbodies. (**b**) Abscission checkpoint signaling in normally segregating cells or cytokinesis with chromatin bridges. Signaling pathways that promote localization of the chromosomal passenger complex (CPC) inside the midbody center are shown in green background, a pathway of Aurora B activation at the midbody center is shown in pink, and signaling pathways that implement the abscission checkpoint downstream of Aurora B are in yellow background. p, phosphorylation. Unknown molecular events are indicated by question marks.

At the midbody, the CPC localizes on the midbody arms in relatively early midbodies (Figure 2a, left; [7,23]). However, as cytokinesis progresses and the microtubule bundles at the midbody gradually become thinner [32,37], a relatively small population of catalytically active Aurora B is detected inside the central region of the midbody (termed “midbody centre” or “Flemming body” [7]), where tubulin staining by immunofluorescence is blocked [23], in late midbodies (Figure 2a, right). This topologically fits well with localization of Aurora B targets (such as Chmp4c) and other abscission-control proteins (such as Vps4 and ANCHR) to the midbody center by confocal microscopy, suggesting this specific part of the midbody can act as signaling hub for the abscission checkpoint [5,6,7]. However, how active Aurora B is recruited to and retained in the midbody center in normally segregating cells or in response to chromatin bridges has remained elusive until recently.

A recent paper identified the DNA double-strand break signaling kinase ataxia-telangiectasia mutated (ATM) and its downstream target checkpoint kinase 2 (Chk2) as regulators of Aurora B recruitment to the midbody center in human cancer cells [81]. ATM and Chk2 localize inside the midbody center in late cytokinesis. Inhibition of ATM or Chk2 impairs CPC localization to the midbody center, accelerates abscission in normally segregating cells and correlates with premature abscission and chromatin breakage in cytokinesis with chromatin bridges. Expression of a chimeric INCENP protein that is specifically targeted to the midbody center rescues premature abscission in Chk2 or ATM-deficient cells in the absence or presence of chromatin bridges, showing that CPC-localization inside the midbody center is required for proper abscission timing [81]. Mechanistically, ATM activates Chk2 in late midbodies. In turn, Chk2 phosphorylates INCENP-serine 91 (S91) to promote stable binding of INCENP to Mklp2 kinesin (Figure 2b, green; [81]). Importantly, while a central region of Mklp2 interacts with INCENP [78,82], the C-terminal part of Mklp2 binds to the central midbody protein Cep55 [81]. Deletion of this C-terminal part of Mklp2 disrupts CPC-localization to the midbody center and accelerates abscission. Furthermore, inhibition of ATM or Chk2 impairs INCENP-S91 phosphorylation inside the midbody center, suggesting that ATM and Chk2 act locally to promote S91 phosphorylation and mediate the binding between the CPC and Cep55-associated Mklp2 in the midbody center (Figure 2b, green).

The Mre11-Rad50-Nbs1 (MRN) complex activates and recruits ATM to broken DNA molecules in the DNA damage response. The MRN localizes to the midbody where it is required for ATM activation in cytokinesis with chromatin bridges, but not in normally segregating cells (Figure 2b, green; [81]). Depletion of MRN proteins accelerates abscission, diminishes CPC-midbody localization and causes chromatin bridges to break [81]. Furthermore, expression of the phosphomimetic S91 to aspartic acid INCENP rescues INCENP localization to the midbody and prevents chromatin breakage in ATM or Mre11-deficient cells. It is proposed that the MRN-ATM-Chk2-INCENP pathway regulates CPC-localization to the midbody through INCENP-S91 phosphorylation, to impose the abscission checkpoint and prevent chromatin breakage in cytokinesis (Figure 2b, green).

The above findings illustrate a fundamental difference in abscission checkpoint activation between budding yeast and human cells: In yeast cells containing Tet operator repeats, forcing Ipl1/Aurora onto chromatin by fusion to the Tet repressor is sufficient to delay abscission independently of anaphase defects, suggesting the CPC acts as a sensor that activates NoCut in response to chromatin in the midzone [8]. In contrast, human cells use a specialized signal transduction pathway to signal chromatin bridges to the CPC, perhaps reflecting differences in the mechanics and regulation of cytokinesis in these species, with budding yeast performing a closed mitosis in which karyokinesis precedes or is tightly coordinated with cytokinesis and mammalian cells undergoing open mitosis [83]. The above findings also raise the more specific question of how chromatin bridges recruit the MRN complex to the midbody in human cells. Additionally, how is ATM activated at the midbody in normal mitosis? Because Aurora B activates ATM in mitosis through ATM-Ser1403 phosphorylation [84], one possibility is that midbody proteins promote or regulate the Aurora B-ATM interaction to regulate abscission timing in normally segregating cells.

### 4.2. Aurora B Activation at the Midbody

Activation of Aurora B requires binding to the IN-box sequence of INCENP and Aurora B autophosphorylation at threonine 232 (T232) within the activation loop of the kinase [85,86], which represents an intermediate state of Aurora B activation [72]. Complete Aurora B activation requires subsequent phosphorylation of INCENP by Aurora B at two consecutive serine residues of a conserved threonine-serine-serine (TSS) motif [71,85] and also phosphorylation of serine 331 (S331) within the Aurora B C-terminal tail, which promotes INCENP-TSS phosphorylation [74]. Interestingly, different kinases mediate Aurora B-S331 phosphorylation throughout mitosis: the DNA damage checkpoint kinase 1 (Chk1) phosphorylates S331 in prometaphase and metaphase [74,87], the structurally unrelated kinase Chk2 mediates S331-phosphorylation in prophase [88] whereas the Cdc-like kinases (Clks) 1, 2 and 4 phosphorylate Aurora B-S331 at the midbody in late cytokinesis [7], perhaps reflecting a requirement for tight spatiotemporal regulation of Aurora B kinase activity in mitosis.

The Clks are evolutionary-conserved dual specificity kinases that regulate alternative splicing through phosphorylating serine/arginine rich domains on splicing factors. Clks 1, 2 and 4 localize to the midbody center as a complex, associate with Aurora B in human cells, and phosphorylate Aurora B-S331 in vitro [7] Inhibition of Clk catalytic activity impairs Aurora B-S331 phosphorylation in late midbodies, accelerates midbody resolution in normally segregating cells, and correlates with premature abscission and chromatin breakage in cytokinesis with chromatin bridges [7]. It is proposed that Clks 1, 2 and 4 phosphorylate Aurora B-S331 inside the midbody center to fully activate Aurora B kinase, to impose the abscission-delay in normally segregating cells or cytokinesis with chromatin bridges (Figure 2b, pink).

## 5. Abscission Checkpoint Signaling Downstream of Aurora B

At the midbody, interaction of the CPC with the ESCRT-III subunit Chmp4c is essential for the abscission checkpoint [5,9]. Chmp4c is dispensable for completion of cytokinesis in normally segregating cells; furthermore, Chmp4c-inhibition accelerates midbody resolution compared with controls, showing that Chmp4c regulates abscission timing [5]. Chmp4c binds to Alix and is recruited to the midbody in an Alix-dependent manner [30,89]. The CPC subunit Borealin also interacts with the N-terminal sequence of Chmp4c; furthermore, Aurora B phosphorylates Chmp4c at serines 210, 214 and 215, inside a C-terminal region that is not shared by the Chmp4a and Chmp4b paralogues, and phosphorylated Chmp4c localizes inside the midbody center in late cytokinesis in control cells [5,7,9]. Inhibition of Aurora B catalytic activity or expression of nonphosphorylatable S210 to alanine (S210A) mutant Chmp4c correlates with mislocalization of Chmp4c to the midbody arms, reduced frequency of cells at the midbody stage in unperturbed mitosis, and with chromatin breakage in cytokinesis with chromatin bridges [5,7]. It is proposed that Aurora B phosphorylates Chmp4c to promote proper Chmp4c-localization inside the midbody center and that this localization is required for the abscission checkpoint (Figure 2b, yellow).

The Abscission/NoCut checkpoint regulator (ANCHR) protein also localizes inside the midbody center [6]. Overexpression of ANCHR delays abscission in normally segregating cells and ANCHR-depletion promotes furrow regression in cytokinesis with chromatin bridges [6]. ANCHR binds to Vps4 ATPase and this association is essential for Vps4-tethering inside the midbody center in late cytokinesis and for the abscission-delay after ANCHR overexpression [6]. Furthermore, ANCHR forms a ternary complex with Chmp4c and Vps4, and Aurora B catalytic activity is required to sustain this complex, presumably through Aurora B-mediated Chmp4c-phosphorylation [5,6]. Taken together, it is proposed that Chmp4c and ANCHR bind to Vps4 inside the midbody center in an Aurora B-regulated manner to delay abscission, perhaps by delaying relocalization of Vps4 from the midbody to the secondary ingression site where it is required for membrane constriction by Chmp4b filaments (Figure 2b, yellow; [6,42]). However, this mechanism is unlikely to fully account for the abscission delay in the presence of chromatin bridges which can last for several hours in wild-type cells.

The Unc-51-like kinase 3 (ULK3) also localizes inside the midbody center and is required for proper abscission timing in normally segregating cells and for the abscission-delay in response to chromatin bridges [90]. ULK3 phosphorylates the ESCRT-III subunit IST1 at specific residues; furthermore, expression of nonphosphorylatable IST1 in which the ULK3-target residues are changed to alanine impairs the abscission delay in the presence of chromatin bridges [90]. It would perhaps be important to examine whether mutating the ULK3-target sites to alanine disrupts IST1-organization into spirals at the intercellular canal [39].

Additionally, a cytoplasmic mechanism that contributes to the abscission delay during conditions that activate the abscission checkpoint was recently identified [91]. Cytoplasmic compartments called “abscission checkpoint bodies” that contain phosphorylated Aurora B-T232, phospho-Chmp4c, Chmp4b and Alix (but, perhaps unexpectedly, not INCENP; [14]), develop after depletion of nuclear pore proteins and their presence correlates with delayed localization of Alix to the midbody and delayed abscission, suggesting these compartments function to restrict recruitment of abscission proteins to the midbody [91]. Although these abscission checkpoint bodies are not detected in the presence of chromatin bridges [91], cytoplasmic mechanisms that contribute to the abscission checkpoint in the presence of DNA bridges remain a possibility.

## 6. Switching the Checkpoint Off: Counteracting Aurora B Kinase Activity

When the abscission checkpoint is satisfied, the Aurora B kinase activity at the midbody is opposed by phosphatases and abscission proceeds. In normally segregating cells, the DNA repair protein Rap1-interacting factor 1 (RIF1) localizes to the midbody center where it recruits protein phosphatase 1 isoform γ (PP1γ; [67]). Depletion of RIF1 or PP1-inhibition delays abscission and increases localization of phosphorylated Chmp4c-S210 and total Vps4 to the midbody [67]. It is therefore proposed that the RIF1-PP1γ complex silences the abscission checkpoint by counteracting the Aurora B-mediated Chmp4c-S210 phosphorylation. Additionally, the protein kinase C-epsilon (PKCε) localizes to the midbody and phosphorylates Aurora B-serine 227 (S227; [92,93]). This phosphorylation alters the Aurora B-substrate specificity as determined by comparing phosphorylation of a peptide array of established Aurora B substrates by wild-type or nonphosphorylatable mutant Aurora B-S227A. Furthermore, PKCε-inhibition or expression of Aurora B-S227A correlate with mislocalization of Chmp4c to the midbody and promote binucleation [92]. Therefore, one possibility is that PKCε switches the abscission checkpoint off by reducing the affinity of phosphorylated Aurora B-S227 for Chmp4c thus leading to Chmp4c-S210-dephosphorylation by PP1 or other phosphatases.

Depletion of PP1β phosphatase or its regulatory subunit myosin phosphatase target subunit 1 (MYPT1) also delays abscission in normal mitosis, perhaps by preventing PP1β-MYPT1-dependent dephosphorylation of Mklp1-serine 708, which is an Aurora B target site [94,95]. Additionally, B56-bound protein phosphatase 2A (PP2A) opposes Aurora B phosphorylation of Mklp2 kinesin at serine 878, inside a lipid association motif [96]. One possibility is that dephosphorylation of Mklp2-S878 by PP2A targets Mklp2 to the plasma membrane and promotes abscission, perhaps by enhancing formation of a densely organized intercellular bridge to generate a stable abscission site [96].

Additionally, the CDK11^p58^ kinase forms a complex with cyclin L1β inside the midbody center [97]. Depletion of CDK11^p58^ delays abscission and reduces formation of Chmp4b filaments at the abscission site in normal mitosis, whereas inhibition of Aurora B activity rescues the above phenotypes. It is proposed that CDK11^p58^ opposes Aurora B activity through an undescribed mechanism, perhaps by promoting recruitment of a counteracting phosphatase to the midbody, to enable abscission [97].

## 7. Stabilization of Chromatin Bridges and Intercellular Canals

The abscission-delay imposed by the abscission checkpoint is not sufficient to prevent chromatin bridge-breakage or cleavage furrow regression during cytokinesis [4,98]. As a result, cells also employ actin polymerization to stabilize chromatin bridges and intercellular canals in the presence of lagging chromatin.

**Figure 3:**
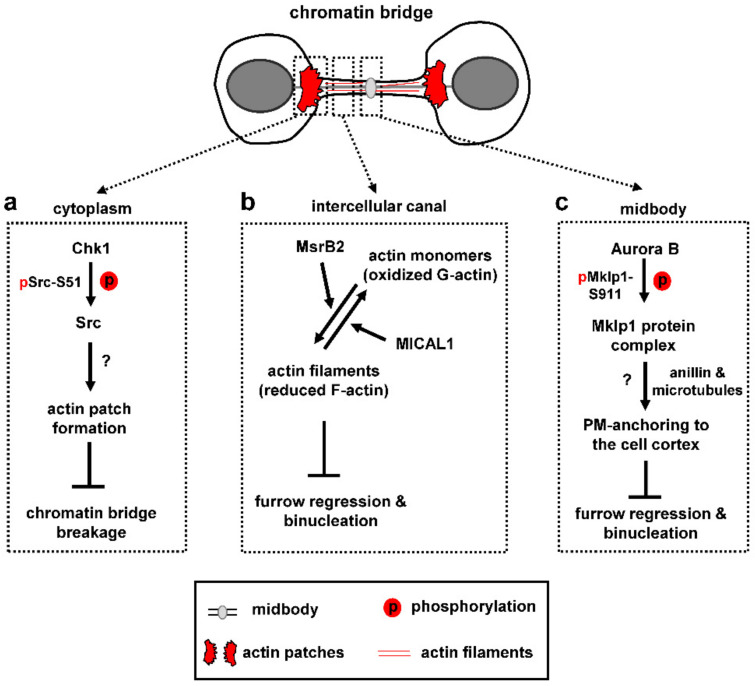
Stabilization of chromatin bridges and intercellular canals in mammalian cells. (**a**) Formation of actin patches at the base of the intercellular canal prevents chromatin breakage. (**b**) MsrB2 reductase prevents depolymerization of actin filaments inside the intercellular canal. (**c**) Potential mechanism that promotes plasma membrane (PM)-anchoring to the cell cortex to prevent binucleation. p phosphorylation. Unknown molecular events are indicated by question marks.

**Figure 4:**
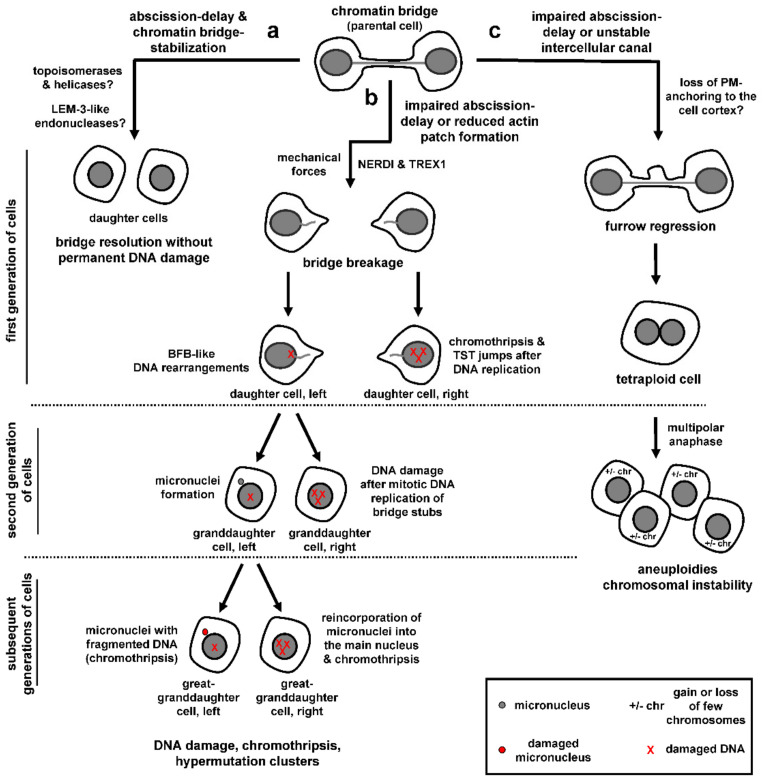
Potential outcomes of cytokinesis with chromatin bridges. (**a**) In abscission checkpoint-proficient cells with stable DNA bridges, chromatin bridges can be resolved without permanent DNA damage. (**b**) Impaired abscission-delay or reduced actin patch formation can cause chromatin bridge breakage, leading to accumulation of DNA damage, chromothripsis and generation of hypermutations clusters. (**c**) Unsuccessful resolution of chromatin bridges can lead to cleavage furrow regression, generation of tetraploid cells and chromosomal instability. See text for details. NERDI, nuclear envelope rupture during interphase; BFB, break–fusion–break; TST, Tandem Short Template; PM, plasma membrane; chr, chromosomes.

Human cells with chromatin bridges form “actin patches”, i.e., accumulations of polymerized actin, at either side of the chromatin bridge which serve to stabilize the chromatin bridge (Figure 3a; [4,98]). Src, a nonreceptor tyrosine kinase that regulates actin remodeling, localizes to actin patches in control cells; furthermore, inhibition of Src catalytic activity correlates with impaired actin patch formation and chromatin bridge-breakage in cytokinesis [98]. Importantly, chromatin breakage in Src-deficient cells is not caused by premature abscission because broken chromatin bridges often exhibit intact intercellular canals; furthermore, expression of a dominant-negative Vps4 mutant that inhibits abscission does not prevent chromatin bridge breakage in these cells [98]. Chk1 is also required for actin patch formation and stable chromatin bridges through an indirect mechanism: Chk1 phosphorylates Src-serine 51 (S51) and this phosphorylation is required for optimal Src-localization and complete Src-catalytic activity [98]. In turn, active Src promotes actin patch formation and stabilizes chromatin bridges in cytokinesis (Figure 3a).

The above findings raise the question of how the presence of chromatin bridges is signaled to Src to generate actin patches. Additionally, the molecular pathways of actin patch formation and the mechanisms by which actin patches prevent chromatin breakage require further investigation. One possibility is that the dense actin filaments network inside the actin patches increases the stiffness of the nuclear envelope and underlying chromatin at the base of the DNA bridge to maintain their integrity [99]. Because focal adhesion proteins involved in Src-signaling (such as the focal adhesion kinase FAK and cortactin) are also detected at actin patches [98], another possibility is that focal adhesions at actin patches may act as “brakes” to reduce the velocity by which the two daughter cells move towards opposing directions thus diminishing the poleward forces exerted on the bridge DNA. Identifying the signaling pathways involved in actin patch formation will be important to further understand how they function.

In the presence of chromatin bridges, cells also prevent the depolymerization of actin filaments inside the intercellular canal that links the two daughter cells (Figure 3b). For this purpose, a nonmitochondrial, cytosolic pool of the human methionine sulfoxide reductase B2 (MsrB2) is recruited to the midbody in response to chromatin bridges and functions within the intercellular canal to promote actin polymerization [100]. Depletion of MsrB2 reduces the levels of polymerized actin within the intercellular canal and increases the frequency of furrow regression and binucleation in cytokinesis with DNA bridges. Furthermore, in normally segregating cells, MsrB2-depleted cells exhibit accelerated abscission, reduced levels of F-actin inside the intercellular canal and increased localization of the ESCRT-III protein Chmp4b to the abscission site. Importantly, F-actin levels are restored to normal in cells depleted of both MsrB2 and MICAL. In vitro time-lapse analysis of depolymerization rates of single actin filaments shows that, while MICAL1 oxidizes actin filaments driving their depolymerization and formation of oxidized monomers, MsrB2 reduces the oxidized monomers thus allowing them to reassemble into filaments [100]. It is proposed that MsrB2 counteracts MICAL function inside the intercellular canal to prevent actin depolymerization in late cytokinesis. In turn, this pool of polymerized actin delays recruitment of ESCRT-III proteins at the abscission site in normally segregating cells, and stabilizes the intercellular canal to prevent binucleation in the presence of chromatin bridges (Figure 3b). MsrB2 colocalizes with the checkpoint components Aurora B and ANCHR at the midbody in cytokinesis with chromatin bridges [100]; however, whether this interaction is essential for MsrB2 recruitment to the midbody and for coupling checkpoint activation with bridge stabilization remains to be established.

Additionally, Aurora B phosphorylates Mklp1-serine 911 (S911) in cytokinesis [101,102]. Impaired Mklp1-S911 phosphorylation after Aurora B-inhibition correlates with binucleation in cytokinesis with chromatin bridges [4], perhaps by disrupting formation of a multiprotein complex containing Mklp1, PRC1, Kif14 and Citron K that links membrane-bound anillin with cortical microtubules around the midbody to anchor the plasma membrane to the midbody cell cortex (Figure 3c; [103,104]).

## 8. Chromatin Bridge Resolution

### 8.1. Successful Bridge Processing

The presence of mechanisms that delay abscission and stabilize the chromatin bridges and intercellular canals in cytokinesis with chromatin bridges raises the question of whether there are active mechanisms of chromatin bridge resolution in wild-type cells, and also whether/how such mechanisms are coupled with the abscission checkpoint. Spontaneous ultrafine bridges from double-stranded DNA catenates gradually diminish in number with anaphase and their resolution requires recruitment of PICH and BLM helicases and topoisomerase activity (Figure 4a; [54,59,105]). Furthermore, the DNA replication and repair protein RIF1 promotes dissolution of nontelomeric, centromere-proximal DNA entanglements through an incompletely understood mechanism that may involve its binding partner protein PP1 phosphatase [67,106,107]. However, whether such mechanisms are effective in “bulky” chromatin bridges in late telophase remains to be established.

The LEM-3 nuclease localizes to the midbody in C. elegans; furthermore, *lem-3* mutant embryos exhibit relatively high rates of chromatin bridges persisting into the next cell division and increased tetraploidization, suggesting that LEM-3 nuclease activity is required for chromatin bridge processing [108]. Additionally, the Aurora B homologue AIR-2 phosphorylates LEM-3 at serines 192 and 194 and this phosphorylation is required for proper localization of LEM-3 to the midbody [108]. It is unclear how the cleaved DNA that is produced after chromatin bridge resolution by LEM-3 is processed/repaired in the following cell cycle. However, cleavage of intertwined DNA at difficult to replicate common fragile sites by nucleases in early mitosis has been described in human cells [109], suggesting that chromatin cleavage by LEM-3-like nucleases may represent a final opportunity for the cell to resolve chromatin bridges with minimal DNA damage that can be dealt with in the next cell cycle (Figure 4a).

### 8.2. Chromatin Bridge Breakage

Unsuccessful processing of chromatin bridges in abscission checkpoint-deficient cells or after impaired formation of actin patches can lead to chromatin breakage and accumulation of DNA damage (Figure 4b; [5,7,81,98]). Additionally, in checkpoint-proficient cells, chromatin bridges generated by dicentric fusion chromosomes or partial depletion of condensin can break after an abscission delay of several hours, as determined by live-cell microscopy [56,110]. Chromatin bridge breakage in checkpoint-proficient cells correlates with bridge elongation because plating human retinal pigment epithelial-1 (RPE-1) cells in relatively short (100 μm) micropatterns that limit bridge extension diminished bridge breakage compared with cells plated on long (300 μm) micropatterns [110]. Furthermore, incubation with small-molecule inhibitors of myosin-activation or actin assembly delayed chromatin bridge breakage compared with controls, suggesting chromatin bridge breakage after prolonged abscission delay is triggered by mechanical forces [110]. The cytoplasmic 3′ exonuclease TREX1 has also been implicated in cleaving chromosome bridges generated by dicentric fusion chromosomes, by using a cell model for telomere crisis [56]. After an abscission delay of several hours, chromatin bridges in these cells undergo nuclear envelope rupture during interphase (NERDI), followed by accumulation of TREX1 across the length of the bridge, generation of single strand DNA and extensive chromatin breakage [56]. Loss of TREX1 delays but does not block bridge breakage [56]. Furthermore, disruption of nuclear envelope integrity by actomyosin pulling forces could allow access of cytoplasmic nucleases, such as TREX1, to bridge DNA raising the possibility that more than one mechanism can contribute to chromatin breakage, perhaps depending on the cell lines and experimental conditions used. Why do chromatin bridges break in abscission checkpoint-proficient cells? One possibility is that, during prolonged abscission delay, actin signaling becomes weakened thus leading to reduced actin patches, destabilization of the chromatin bridge and bridge-breakage by pulling forces and/or TREX1 (also see previous section “Stabilization of chromatin bridges and intercellular canals”). Another possibility is that, after prolonged activation, cells can escape the abscission checkpoint through a yet unrecognized “adaptation” process in which cells continue to proceed through the cell cycle despite not having resolved their DNA bridges.

Regardless of how chromatin bridges break, chromatin breakage can have catastrophic consequences for genome stability. First, bridge breakage can initiate breakage–fusion–bridge (BFB) cycles that generate gene amplification over multiple cell generations [111,112]. Using live-cell imaging to track bridge chromosomes over two generations of cells followed by single-cell sequencing, Umbreit et al. showed that, in the interphase immediately after the chromatin bridge breaks, the resulting chromatin stubs are incorporated in the daughter nuclei and the majority of daughter cells exhibit relatively simple DNA rearrangements and gene-copy number alterations localized near the sites of DNA breakage that are consistent with the BFB cycle model (Figure 4b, left daughter cell; [110]). Furthermore, a minority of cells derived from bridge breakage (4/20 cells tested) exhibit chromothripsis [111], i.e., clusters of localized chromosome rearrangements from the same or different chromosomes that are then randomly reassembled by DNA repair pathways or aberrant DNA replication mechanisms (Figure 4b, right daughter cell; [113,114]). The above cells also exhibit multiple short (~200 bp) insertions called “Tandem Short Template” (TST) jumps that are present in tandem within rearrangement junctions [110]. It is proposed that TST jumps and chromosome rearrangements after the first mitosis are generated by template-switching errors during DNA replication in a percentage of cells with broken DNA bridges.

When cells with broken bridges enter the following mitosis, the stubs of broken chromosome bridges undergo a burst of mitosis-specific DNA replication (as evidenced by EdU-labeling on the bridge DNA) which associates with generation of DNA damage by γ-H2AX staining (Figure 4b, right granddaughter cell; [110]). The mechanism of triggering mitotic DNA replication on bridge stubs is unknown: Because chromatin bridges have an altered nuclear envelope compared to primary nuclei in the same cell [56], one possibility is that bridge DNA is incompletely replicated during interphase due to abnormal import of key cytoplasmic proteins. Nuclear envelope breakdown in the second mitosis might then trigger mitotic replication of the unreplicated DNA, followed by replication fork collapse and DNA damage. This scenario is reminiscent of micronuclei that share common nuclear envelope defects with chromatin bridges and can exhibit DNA damage [56,115,116]. Furthermore, after the second mitosis, approximately 50% cell divisions produce cells with micronuclei that contain bridge DNA (Figure 4b, left granddaughter cell; [110]). This may be caused by chromatin breakage impairing centromere or kinetochore functions and can initiate rounds of chromothripsis at the micronuclei (Figure 4b, left great-granddaughter cell). Furthermore, chromosomes within micronuclei can reincorporate into the primary nucleus at subsequent cell divisions leading to extensive chromosome rearrangements inside the main nucleus (Figure 4b, right great-granddaughter cell; [117,118,119]). Consistently, bulk whole-genome sequencing on subclones derived from single cells that are isolated after initial bridge formation and breakage shows complex chromosomal rearrangements (chromothripsis) and localized hypermutation clusters (“kataegis”) close to the genomic breakpoints, i.e., groups of C to T and C to G base substitutions at TpC dinucleotides that are associated with APOBEC (apolipoprotein B mRNA editing enzyme, catalytic polypeptide-like) family-mediated cytosine deamination and are widespread in human cancers [56,111,120,121,122].

### 8.3. Cleavage Furrow Regression

Impaired abscission checkpoint signaling or inefficient stabilization of the intercellular canal may also lead to cleavage furrow regression and generation of tetraploid cells, perhaps due to loss of plasma membrane anchoring to the cell cortex (Figure 4c; [4,6,100]). Tetraploid cells are normally eliminated from the replication pool by apoptosis or replicative senescence in a P53-dependent manner; however, in the absence of functional P53 protein, they can undergo multipolar anaphase with multiple cleavage furrows resulting in aneuploidy with loss or gain of few chromosomes and chromosomal instability (Figure 4c; reviewed in [12]). Why impaired abscission checkpoint signaling leads to chromatin bridge breakage in some cases [5,7,81] but furrow regression in others [4,6] remains unclear and could depend on the experimental conditions used [18]. In conclusion, improper resolution of chromatin bridges in checkpoint-deficient cells can lead to profound genomic alterations such as tetraploidization, chromosomal BFB events or chromothripsis, which are associated with cancer development or progression [123,124,125,126].

## 9. Perspectives

Work in the last decade has demonstrated that impaired abscission checkpoint signaling can lead to genome aberrations and chromosomal instability (CIN), which are associated with carcinogenesis [127]. Genomic indicators of chromosome BFB cycles, chromothripsis or kataegis that can result from inappropriate chromatin bridge resolution are detected in a variety of human cancers, thus supporting a connection between abscission checkpoint defects and cancer development or evolution [122,125,128,129,130]. Overexpression of Aurora B has also been detected in several tumour types and is associated with unfavorable prognosis for cancer patients [131]. Additionally, a naturally occurring human Chmp4c^T232^ polymorphism encoding an amino acid substitution of Chmp4c-alanine 232 to threonine that does not bind to Alix, exhibits impaired abscission delay in cytokinesis with DNA bridges and associates with increased susceptibility to ovarian cancer [13,132]. Although expression of the *chmp4c^T232^* risk allele could also impair other cell functions such as the mitotic spindle checkpoint and chromosome segregation [133,134], together the above findings support a protective role for the abscission checkpoint against tumourigenesis.

Can the abscission checkpoint be employed for cancer therapy? A role for CIN in stimulating carcinogenesis by generating the genetic diversity that is required for cancer formation or adaptation is well established [127]. However, at least in certain cases, excessive CIN can be poorly tolerated by cancer cells, perhaps because a critical limit that is compatible with cancer cell survival and fitness is exceeded [135,136]. Furthermore, excessive CIN may generate “synthetic lethal” interactions specifically in tumours, by inducing gene dependencies that are absent from normal cells [136,137,138]. As a result, pharmacological inhibition of abscission checkpoint proteins could increase genomic instability and selectively target chromosomally unstable cancer cells while being potentially less toxic for normal tissues. Such abscission checkpoint-inhibitors could be administered as monotherapy or in combination with other anticancer treatments to enhance tumour cell-killing; furthermore, several inhibitors of Aurora B kinase are now tested in clinical trials [131]. Perhaps supporting targeting abscission-control proteins for cancer therapy, the Chmp4c^T232^ mutation sensitizes cancer cells to replication stress by low doses of the DNA replication inhibitor aphidicolin and synergizes with loss of p53 [13]. Furthermore, depletion of Chmp4c sensitizes a human lung cancer cell line to killing by gamma irradiation [139]. Additionally, expression of a non-phosphorylatable S91A mutant INCENP that does not localize to the midbody center and is impaired for the abscission checkpoint diminishes cell proliferation [81]. Further understanding of the molecular mechanisms by which the abscission checkpoint guards against chromosomal instability will help us devise new ways of exploiting the abscission checkpoint to improve cancer therapy.

## Data Availability

Not applicable.
